# LY6E as a new prognostic biomarker of multiple myeloma-related bone disease

**DOI:** 10.1038/s41598-025-91413-1

**Published:** 2025-04-03

**Authors:** Min Shi, Jing Li, Jing Wang, Ye Yao, Xuxing Shen, Yuan Xia, Ji Xu

**Affiliations:** 1 Department of Hematology, Jiangsu Province Hospital, The First Affiliated Hospital of Nanjing Medical University, Nanjing, 210029 China; 2 Department of Hematology, the First Affiliated Hospital of Anhui University of Chinese Medicine, Hefei, 230601 China

**Keywords:** LY6E, Multiple myeloma, Bone disease, Osteoclast, Myeloma, Haematological cancer

## Abstract

Osteolytic bone disease, which deteriorates the quality of life, is a prevalent complication of multiple myeloma (MM). In this study, we utilized bioinformatics analysis to identify the differentially expressed genes (DEGs) associated with MM bone disease (MBD) from the Gene Expression Omnibus (GEO) databases. Here, the Kaplan-Meier (K-M) curve and Cox regression analyses demonstrated that the key molecule lymphocyte antigen 6 complex (LY6E) was closely correlated with the MM progression, unfavorable prognosis and the formation of MBD. Furthermore, we confirmed that higher LY6E expression promoted MM cell proliferation and osteoclast differentiation in vitro. Taken together, these findings may illuminate the theoretical foundation for LY6E in MBD formation and identify it as a neoteric therapeutic target for MM.

**Supplementary Information:**

The online version contains supplementary material available at 10.1038/s41598-025-91413-1.

## Introduction

Multiple myeloma (MM) is a cancerous proliferation of monoclonal plasma cells^1–4^, and the incidence of MM has increased to 18.7% of hematological malignancies in the USA. MM bone disease (MBD) is a hallmark of MM, and 80% of newly diagnosed MM (NDMM) patients have osteolytic lesions^5^. Furthermore, MM patients presenting with MBD have a higher risk of skeleton-related events (SREs) such as pathological fractures and spinal cord compression. Unlike most solid tumors, bone destruction caused by MM is characterized as lytic lesions^6–8^. MM cells secrete the growth factors impacting the activity of osteoblasts and osteoclasts, leading to an uncoupling between bone resorption and formation. This process establishes abnormal bone remodeling and perpetuates a continuous vicious cycle^9–11^. Consequently, osteoclast proliferation is enhanced and osteogenesis is suppressed. MBD adversely influences the quality of life and poor prognosis in MM patients, but its ontogenesis and vulnerabilities remain unclear^12^.

Potential therapeutic targets associated with bone destruction require further identification and exploration. The receptor activator of nuclear factor-kappa B ligand (RANKL) plays a crucial role in osteoclast formation, and the RANK/RANKL pathway is pivotal in bone remodeling. Recent studies have demonstrated that RANKL expression is expressed in MM patients and myeloma cells^13–15^. However, the survival of tumor cells is not only directly affected by RANKL, but also by the significant elevation TNF-α, IL-6, and IL-8 induced by RANKL^16,17^. Several studies reported that typical Wnt signaling exerted a dual effect in MM-related bone disease. It was involved in the proliferation, migration, and drug resistance of MM, as well as the secretion of Wnt antagonist DKK1, which inhibited osteoblast differentiation and promoted the development of lytic osteopathy^18,19^. Additionally, Sebag et al.^20^ found CCL-3/MIP-1α as a chemokine that stimulated osteoclasts and was associated with the extent of lytic bone lesions observed in patients with MM.

Thus far, bisphosphonates and a RANKL inhibitor denosumab have been established to reduce bone destruction, SREs and prolong the survival of patients conclusively^21–23^. However, the adverse effects of these drugs such as renal toxicity, secondary mandibular osteonecrosis, and hypocalcemia should not be ignored. To date, there is no pharmaceutical agent that can effectively address the underlying pathogenesis of MBD. The available therapies merely mitigate the bone-destructive process. Therefore, it is of great clinical value to in-depth study MBD, further elucidate its formation mechanism and explore the key molecules. Here, we integrated Gene Expression Omnibus (GEO) datasets, analyzed clinical data and combined experiments in vitro. Our study demonstrated that MM bone-associated prognostic gene *LY6E* promoted MM cell proliferation and induced osteoclast generation. These results indicated that LY6E was of paramount importance in the occurrence of MM and the formation of bone lesions, which might be a prognostic biomarker for MM.

## Materials and methods

### Gene expression profiling and patient samples

This study encompassed five independent gene expression profiling datasets, including the training dataset GSE2658 (comprising 559 MM samples), the validation dataset GSE24080 (encompassing the clinical characteristics of 559 MM samples), GSE6477 (containing 162 samples), GSE9782 (containing 264 samples), and GSE5900 (containing 78 samples). The samples in GSE2658 and GSE24080 were CD138 + plasma cells derived from bone marrow. The R-package “limma” was employed for the screening of differentially expressed genes (DEGs), with a threshold of |logFC|>1.5 and a *P-*value < 0.05. All relevant clinical information on MM patients is publicly accessible via the GEO database (http://www.ncbi.nlm.nih.gov/geo).

### Functional enrichment analysis

The R-package “clusterProfiler” was utilized to analyze Gene Ontology (GO) terms and Kyoto Encyclopedia of Genes and Genomes (KEGG) pathways^24^. GroupGO is a gene classification method that is employed to enrich the GO terms of molecular function (MF), biological process (BP), and cellular component (CC). In addition, gseKEGG was enriched by the KEGG pathway^25,26^.

### Protein-protein interaction (PPI) network construction and modular analysis

The PPI network was used to evaluate the DEG-encoded proteins and PPI information was acquired from the STRING database (http://string-db.org). Cytoscape software version 3.8.0 was applied to establish the network. MCODE, a plug-in Cytoscape, was utilized to screen the modules from the PPI network and determine the most significant module based on the MCODE score and node number.

### Prognostic predictive value of LY6E

We used univariate and multivariate Cox regression models to identify the level of LY6E expression and the clinical features associated with survival. When predicting prognostic value, incomplete survival time and state of MM samples were excluded. The cut-off value of LY6E for risk-predictive classification was determined based on survival status at a 2-year time point using the “survminer” package. The MM samples were subsequently categorized into high- and low-expression groups according to whether their LY6E scores exceeded or fell below this cut-off value. Kaplan-Meier (K-M) curves were plotted to perform the overall survival (OS) and event-free survival (EFS) in GSE2658, GSE24080 and GSE9782 using the R packages “survival” and “survminer”. In the correlation analysis with clinicopathologic characteristics of MM patients, samples designated as “unknown”, “bland”, and others were excluded.

### Immunohistochemistry (IHC) assay

All NDMM patients were diagnosed in accordance with the criteria of Chinese Guidelines for the Diagnosis and Treatment of multiple myeloma. Bone marrow biopsy tissues from NDMM patients were selected for paraffin embedding. The slides were incubated with primary anti-LY6E (ab300399, abcam) at 4℃ overnight. On the second day, the slides were treated with secondary antibody at room temperature for 2 h. After staining with diaminobenzidine (DAB), the slides were counterstained with hematoxylin. LY6E IHC staining was conducted by the following staining intensity scores: 0, negative; 1+, weak; 2+, moderate; and 3+, strong. The final IHC score was obtained by multiplying each intensity score multiplied by the proportion of stained cells. The immunohistochemical samples were obtained from the First Affiliated Hospital of Nanjing Medical University (ethics number: 2021-SR-319). All experiments were conducted following relevant guidelines and regulations. The authors complied with the ARRIVE guidelines.

### Cell lines and culture

Human MM cell lines MM.1 S (dexamethasone sensitive), U266, OPM2 and RPMI-8226 were cultured in RPMI-1640 medium (Gibco, USA). The RAW264.7 cell lines were maintained in DMEM. MC3T3-E1 cells were cultivated in α-MEM (Gibco, USA). All the cells were sponsored by Prof. Yongqiang Zhu (Nanjing Normal University, China). The medium was added in 10% fetal bovine serum (FBS, Gibco, USA) and 1% penicillin/streptomycin (Biosharp, China) under a 5% CO_2_ atmosphere at 37 °C.

### Cell viability detection

MM cell lines (5 × 10^3^ cells per hole) were inoculated in 96-well plates. Cell viability was measured daily using the Cell Counting Kit-8 (CCK-8) (Biosharp, China) over 96 h. Absorbance was checked at 450 nm using a microplate reader (Thermo Fisher Scientific) after adding CCK-8 reagent to each hole for 2 h.

### Plasmid and cell transfection

Two sequence LY6E-shRNA were cloned into pLKO.1-EGFP-puro by AgeI-EcoRI (Genebay, China). The LY6E-FLAG sequence was cloned into pCDH-CMV-MCS-EF1-CopGFP-T2A-Puro (CD513B) using EcoRI-BamHI. Lipofectamine 3000 reagent (Invitrogen, USA) was used for transfection. With GFP shRNA as a control group, lentivirus was generated in 293T cells by cotransfection of pMD2.G and psPAX2 vectors. At 48 h after transfection, we refreshed the culture medium and concentrated the virus. OPM2 or RPMI-8226 cells and U266 or MM.1 S were infected with lentivirus containing LY6E shRNA and LY6E-FLAG, respectively. Finally, the transfection efficiency was verified by Western blot assay.

### RNA extraction

The total RNA of the osteoclast was extracted using TRIzol reagent (Invitrogen, CA, USA). Reverse transcription of RNA was accomplished by Super Script II reverse transcriptase (Vazyme, Nanjing, China). The results were standardized with the GAPDH gene. Primers were listed in Supplementary Table S1.

### Osteoclast and osteoblast differentiation

Raw264.7 cells were inoculated into 6 × 10^4^ cells/well of a 12-hole well plate. At 24 h post-starvation, the supernatant of MM cells was used as the conditioned medium (CM). The osteoclast medium consisted of DMEM incubated with 15ng/ml RANKL (R&D Systems, USA) and CM (DMEM: CM = 1:1) and was changed every 2 days. When multicellular osteoclasts were observed on d8, tartrate resistant acid phosphatase (TRAP) staining was performed (Servicebio, China) and osteoclast differentiation was observed. MC3T3-E1 (6 × 10^5^ cells per well of a 6-hole plate) cells were seeded in CM and differentiation medium (α-MEM consisting of 10% FBS and 1% penicillin/streptomycin, 10mM β-glycerophosphate [Sigma], 100 nM dexamethasone [Sigma], and 200 µM L-ascorbic acid [Fisher chemical]). Alizarin red S staining was performed on d21.

### Statistical analysis

Experiment results were performed by R software v. 4.0.3 and GraphPad Prism (V.6.0). The values are expressed as the mean ± S.D. The analysis of numerical variables was performed by *t* test or analysis of ANOVA, while the analysis of non-normal distribution variables adopts the Mann-Whitney U test. The survival curve was plotted and analyzed by the K-M curves and the Log-rank test. Univariate and multivariate analysis were performed using the Cox regression model. *P* < 0.05 was defined as a statistical difference. All experiments were repeated at three times.

## Results

### The overall design of the study

**Fig. 1:**
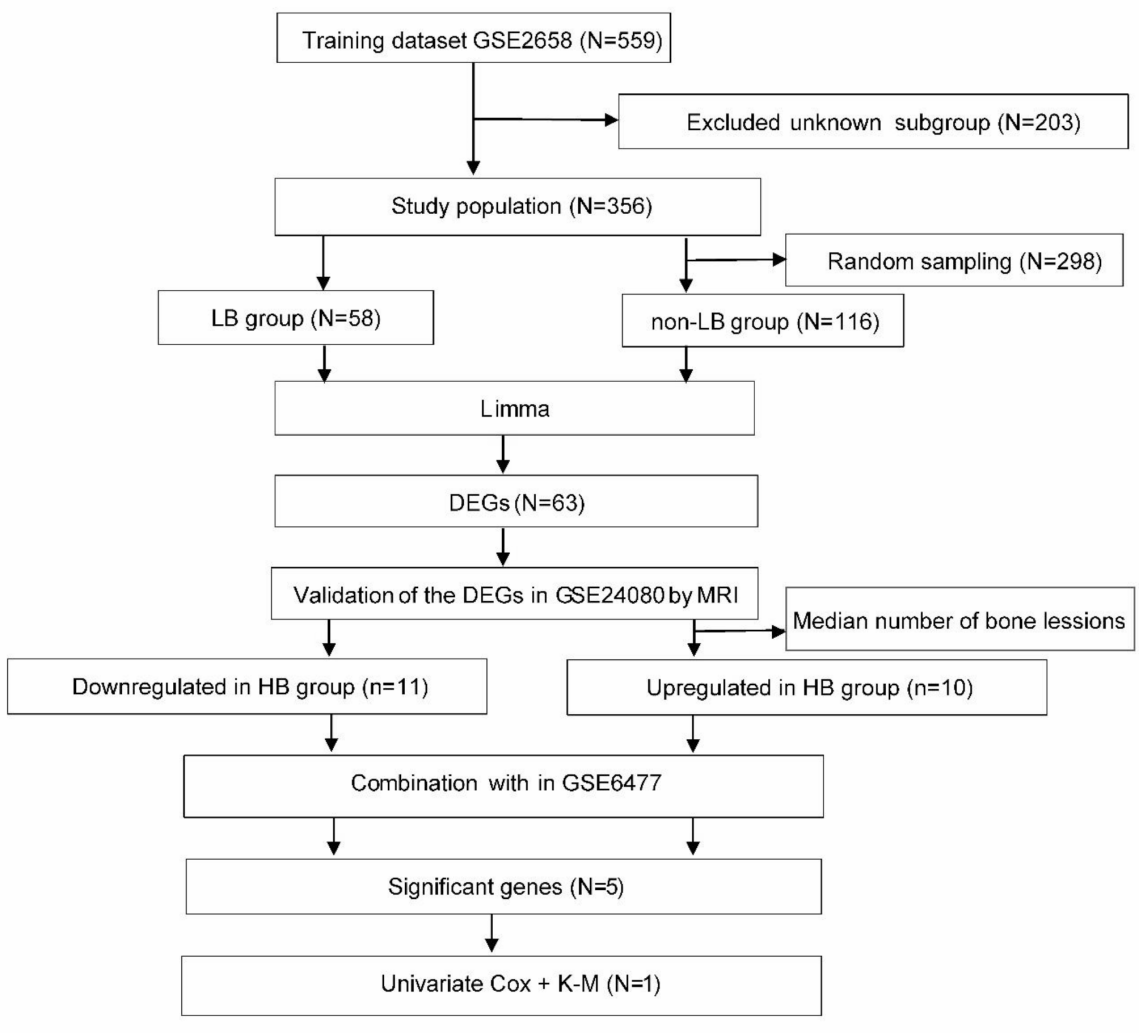
The design to obtain and verify the prognostic gene of MBD.

A total of 559 MM samples were obtained from GSE2658, including mRNA expression and clinical information. Zhan et al. divided the gene detection of the new diagnosis MM (NDMM) into seven categories: LB (low bone disease), PR (proliferation), HY (hyperdiploid), MS (MMSET), MF (MAF/MAFB), CD1 and CD2 (spiked expression of CCND1 and CCND2)^27^. We split the GSE2658 dataset into two groups: the LB group and the non-LB group (the other six subgroups). The LB group comprised the whole of 58 samples, whereas the non-LB group consisted of 116 samples. These samples were randomly selected in a 1:2 ratio. As shown in (Figs. 1), 63 bone-associated DEGs were obtained based on the standard of |logFC|>1.5 and *P* < 0.05. To validate the DEGs, we used the clinical information including magnetic resonance imaging (MRI), survival time and status in GSE24080. Among the above 63 DEGs, 21 (11 upregulated and 10 downregulated genes) genes maintained their value in the high bone disease (HB) group. At the same time, we utilized the GSE6477 to compare the gene expression in normal people and MM patients. After intersecting with the above 21 DEGs, the 5 DEGs were obtained in the MM group. The survival analyses of these 5 DEGs revealed that *LY6E* serves as an independent biomarker for poor PFS and OS. Hence, the *LY6E* gene was associated with MBD and MM survival.

### Identification of DEGs in GO and KEGG pathway enrichment analyses

**Fig. 2:**
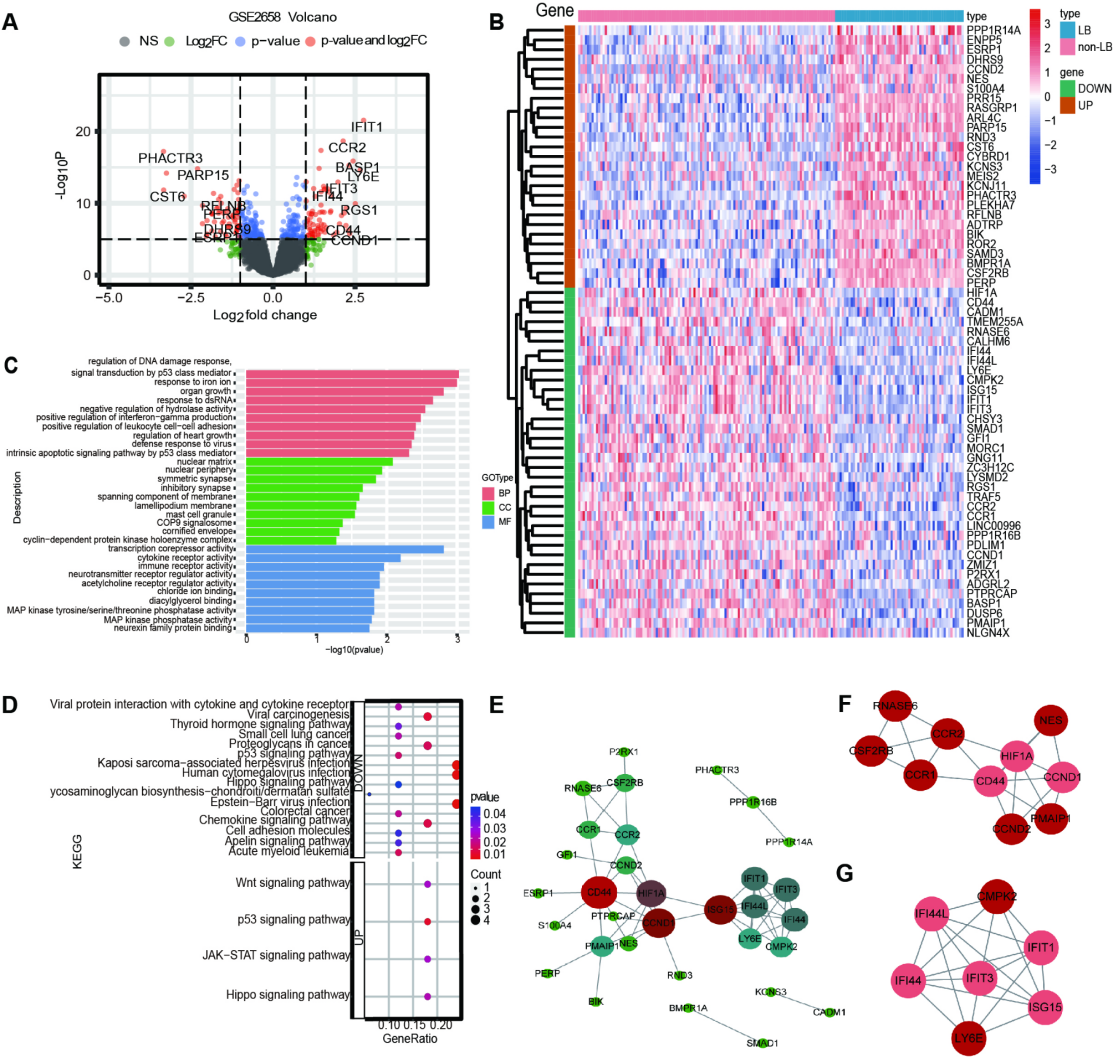
DEGs and functional enrichment analysis in GSE2658. (**A**)Volcano plot and (**B**) heatmap of the 63 DEGs. The plot of (**C**) GO enrichment and (**D**) KEGG pathway among DEGs. The PPI network of MBD-related genes and top 2 clusters with higher scores from PPI. (**E**) 32 nodes (meant the DEGs) and 55 edges (meant the link between DEGs) were exhibited. As the increased degree, dot color changed from green to red, dot size from small to large. The large size and red color of the nodes indicated that they the nodes were connected to other nodes. (**F**) 10 nodes in Cluster 1and (**G**) 7 nodes in Cluster 2 (dark red meant a high score, the pale red meant a low score).

To effectively distinguish MBD-related genes, 63 DEGs were shown as the volcano plot and heatmap in GSE2658 (Fig. 2A, B). Compared with the LB group, 27 genes were downregulated and 36 genes were upregulated in the non-LB group. To figure out the biological function of DEGs, the GO and KEGG analyses were performed by clusterProfiler from R 4.0.3. GO enriched in three aspects: BP, CC, MF, and the top ten pathways were plotted (Fig. 2C). The BP group showed that the DEGs were enriched in the regulation of DNA damage including p53 class mediator, responsive to iron, dsDNA and positive regulation of interferon-gamma production. For CC, they were enriched in cell nuclear, cell membrane, and synapse. Towards MF, the DEGs were mainly enriched in transcription corepressor, cyclin, and immune activity. Moreover, the upregulated DEGs were enriched in four signaling pathways in KEGG enrichment: Wnt, p53, JAK-STAT, and Hippo pathways. However, most pathways were enriched in viral protein interaction with cytokine and cytokine receptors, chemokine signaling pathway, and cell adhesion molecules in downregulated DEGs (Fig. 2D).

### Regulation of DEGs in the PPI network

To gain a deeper insight into the protein interaction among these DEGs, a PPI network was constructed, which revealed 32 nodes and 55 edges in myeloma cells, comprising 12 up-regulated and 20 down-regulated genes (Fig. 2E). Two clusters were selected through the MCODE scores: Cluster 1 contained 10 nodes and 19 edges (Fig. 2F), while Cluster 2 consisted of 7 nodes and 18 edges (Fig. 2G). The new perspective revealed that *CD44*, *HIF1A*, *CCND1*, *LY6E*, and *CCR1* were strongly linked with other genes across three PPI networks. To sum up, these findings indicated that *LY6E* may be significantly associated with interferon family-related genes.

### DEGs in GSE24080 and GSE6477

**Fig. 3:**
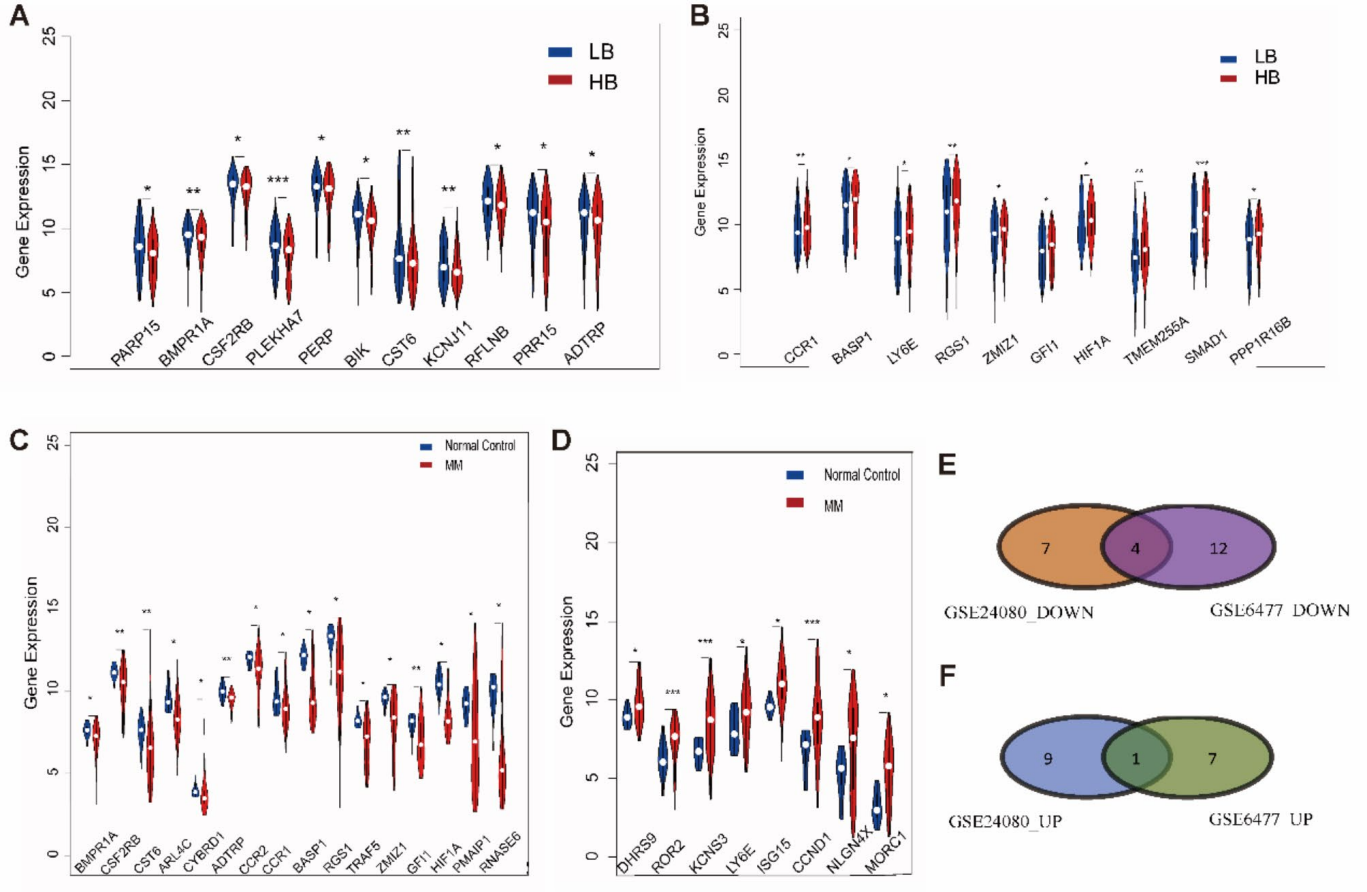
Screening of MBD-related genes. (**A**) 11 genes were low expressed in the HB group of GSE24080. (**B**) 10 genes were highly expressed in the HB group of GSE24080. (**C**) 16 genes with low expression in the MM group of GSE6477. (**D**) 8 genes increased in the MM group of GSE6477. (**E**) The Venn diagram of the genes in GSE24080_DOWN and GSE6477_DOWN. (**F**) Venn diagram of the genes in GSE24080_UP and GSE6477_UP. * *P* < 0.05; ** *P* < 0.01; *** *P* < 0.001.

Based on the median number 3 of bone lesions observed in the MRI, we divided the MM samples into the LB (MRI < 3 lesions) and HB (MRI ≥ 3 lesions) groups in GSE24080. Among the 21 DEGs, 11 genes including *ADTRP*, *PRR15*, *RFLNB*, *KCNJ11*, *CST6*, *BIK*, *PERP*, *PLEKHA7*, *CSF2RB*, *BMPR1A*, and *PARP15* were significantly downregulated in the HB group (Fig. 3A). Conversely, 10 genes including *LY6E*, *BASP1*, *CCR1*, *GFI1*, *HIF1A*, *RGS1*, *PPP1R16B*, *SMAD1*, *TMEM255A*, and *ZMIZ1* demonstrated increased expression in the HB group (Fig. 3B). Subsequently, the DEGs were incorporated into the analysis by comparing the normal control and MM groups in GSE6477. The results demonstrated that 16 genes exhibited low expression levels (Fig. 3C), while 8 genes displayed high expression levels (Fig. 3D) in the MM group. A total of five DEGs were identified as common in the GSE24080 and GSE6477 databases. These included *BMPR1A*, *CSF2RB*, *CST6*, *ADTRP*, exhibited decreased expression, and *LY6E*, which was upregulated (Fig. 3E, F). These findings confirmed that *LY6E* may be a high-risk gene for MBD.

### Analyzing the prognostic impact of LY6E

**Fig. 4:**
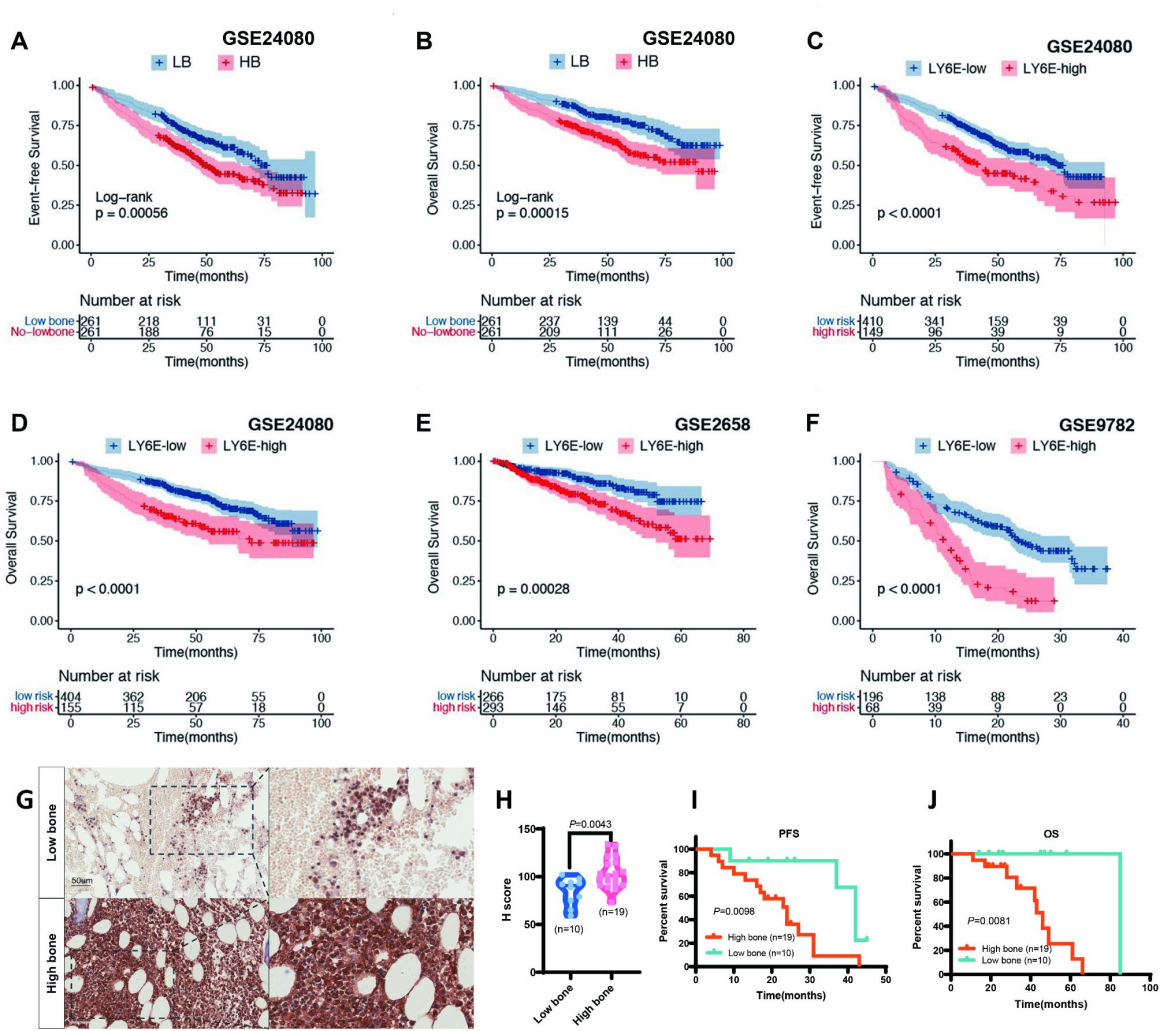
LY6E is related to MM prognosis. (**A**, **B**) K-M curves of EFS and OS among MM patients between the LB and HB groups. (**C**, **D**) K-M curves show the effect of the LY6E on the survival time of MM patients in GSE24080, (**E**) GSE2658, and (**F**) GSE9782 datasets. (**G**-**H**) Representative IHC images for LY6E in low bone and high bone new diagnosed MM patinets. Scale bars, 50 μm. (**I**, **J**) K-M survival curves of PFS and OS in low bone and high bone new diagnosed MM patinets. * *P* < 0.05; ** *P* < 0.01; *** *P* < 0.001.

The aforementioned 5 genes were found to have the potential to predict bone destruction in MM patients. The K-M curve showed that the LB group exhibited superior survival compared to the HB group (*P* < 0.01, Fig. 4A, B) in GSE24080. To determine which genes have prognostic value in MM patients, we employed univariate Cox regression analysis. The results proved that the expression of LY6E was significantly negatively correlated with the prognosis (OS: *HR* = 1.12, 95% *CI*: 1.04–1.20, *P* < 0.01; EFS: *HR* = 1.08, 95% *CI*: 1.02–1.15, *P* < 0.01, Table S2). A survival analysis was conducted on the LY6E in the GEO-MM dataset using three databases. The results showed thar the LY6E-high group exhibited a shorter OS and EFS than the LY6E-low group in GSE24080 (*P* < 0.01, Fig. 4C, D). In GSE2658 and GSE9782, the OS of patients in the LY6E-high group was also significantly shorter than that of patients in the LY6E-low group (Fig. 4E, F). Subsequently, univariate and multivariate Cox regression analysis was carried out to assess the relationship between LY6E and clinical features in GSE24080. A significant reduction in both EFS (*HR* = 1.71, 95% *CI*: 1.31–2.24, *P* < 0.01, Table S3) and OS (*HR* = 1.93, 95% *CI*: 1.41–2.64, *P* < 0.01, Table S4) was observed in the LY6E-high group in comparison to the LY6E-low group.

Further analysis of LY6E expression in plasma cell disease revealed that the combined data from GSE5900 and GSE2658 indicated the highest expression level of LY6E in MM groups. However, the *P*-value could not be obtained due to the smaller sample size of the other groups compared to the MM group (Figure S1A). In addition, the expression of LY6E was found to have increased significantly in patients with relapsed MM in comparison to the baseline in GSE6477 (Figure S1B). To further investigate the result of our center, we randomly recruited 29 NDMM patients and divided them into the LB group (*n* = 10) and the HB group (*n* = 19). Based on the cutoff criteria of the number of two bone lesions in positron emission tomography-computed tomography (PET-CT), the results showed that the relative LY6E expression in bone marrow biopsy tissue IHC of LB group was lower (*P* = 0.043) (Fig. 4G, H). In addition, higher bone lesions were related to diminished PFS and OS (Fig. 4I, J). These data strongly suggested that LY6E expression, which correlated with MBD, increased progressively with the progression of MM disease.

### LY6E promoted MM cell proliferation in vitro

**Fig. 5:**
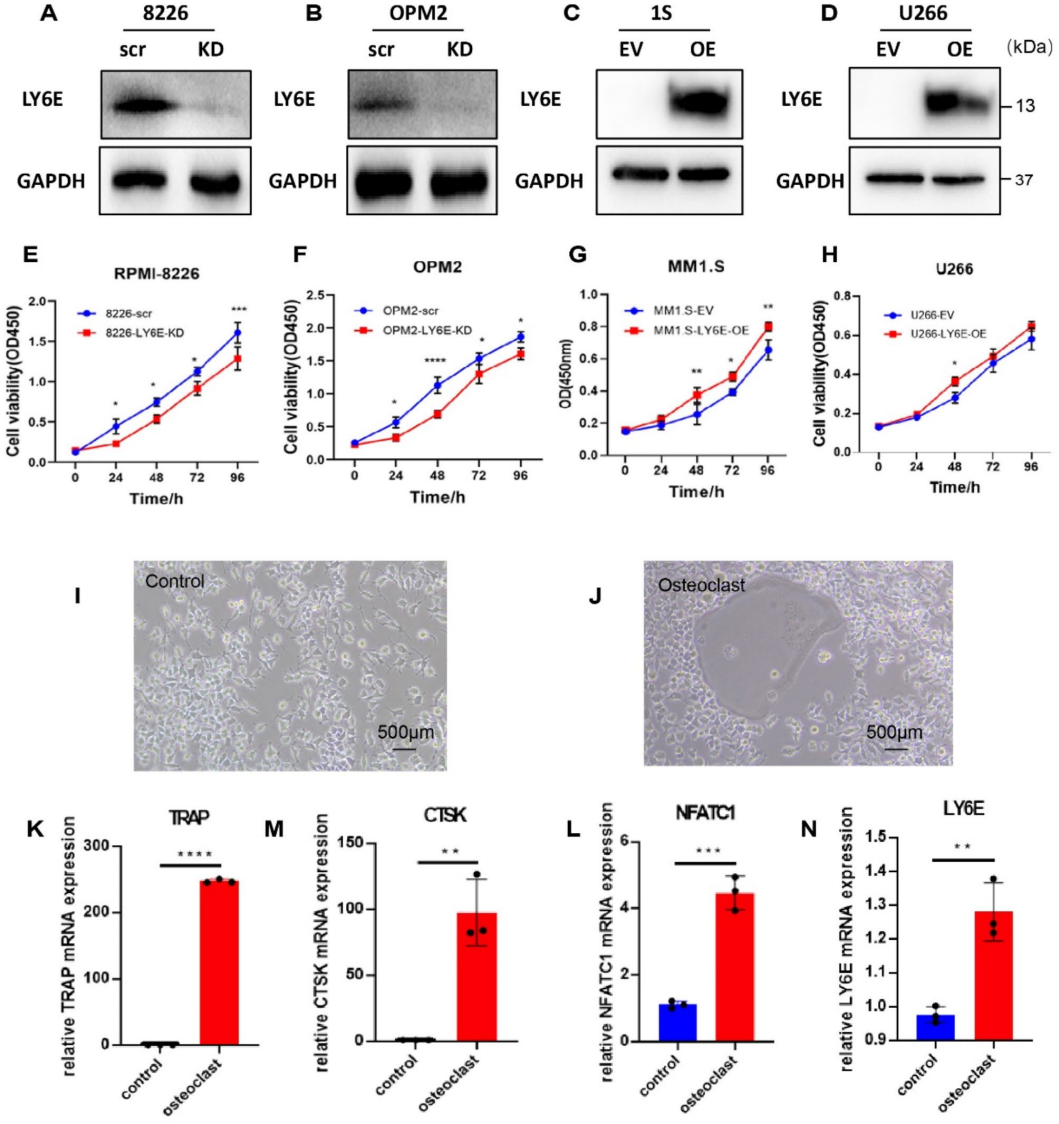
Increased LY6E expression facilitated MM cell proliferation and osteoclast differentiation. (**A**-**D**) Cropped western blots were analyzed the expression of LY6E in LY6E-KD and LY6E-OE in MM cells. Original blots are presented in supplementary Figure S2. (**E**-**F**) CCK-8 was assessed for the reproduction of LY6E-scr and LY6E-KD cells. (**G**-**H**) CCK-8 assay was assessed for reproduction of LY6E-EV and LY6E-OE cells. (I-J) RANKL (15ng/mL) stimulated RAW264.7 to differentiate into osteoclast cells. After 5 days, TRAP staining was performed when the nuclears of osteoclast were over 3 (scale bar = 500 μm). (**K**-**N**) The control and osteoclast groups set the TRAP, CTSK, NFATC1, and LY6E mRNA expression. * *P* < 0.05; ** *P* < 0.01; *** *P* < 0.00.

To investigate whether LY6E could modulate MM cell proliferation, we successfully knocked down LY6E in RPMI-8226 and OPM2 cells and overexpressed LY6E in U266 and MM1.S cells. The transfection efficiency was verified by Western blot (Fig. 5A-D). The CCK-8 assay showed that the proliferation activity of RPMI-8226-KD and OPM2-KD MM cells was significantly inhibited in a time-dependent manner, whereas it was enhanced in MM1.S-OE and U266-OE cells (Fig. 5E-H).

### The role of LY6E in osteoclast formation

In light of our previous dataset research which demonstrated an association between LY6E and bone lesions, we initially investigated the expression levels of LY6E in an in vitro model of bone resorption. RAW264.7, a murine-derived monocyte cell line, was incubated with RANKL (15ng/mL) to induce osteoclast differentiation. The formation of osteoclasts was demonstrated by TRAP staining on d5 (Fig. 5I-J). The classical markers of osteoclast differentiation-related gene TRAP, cathepsin K(CTSK), and nuclear factor of activated T-cells cytoplasmic 1(NFATC1) mRNA exhibited a notable elevation on d4 (Fig. 5K-L), which indicated that our osteoclast model was suitable for further analysis. Besides, the expression of LY6E was observed to increase during the process of osteoclast differentiation (Fig. 5N). Therefore, it can be proposed that LY6E exerts a promoting influence on osteoclast activity in bone metabolism.

### LY6E-induced osteoclast differentiation and function in MM cells

**Fig. 6:**
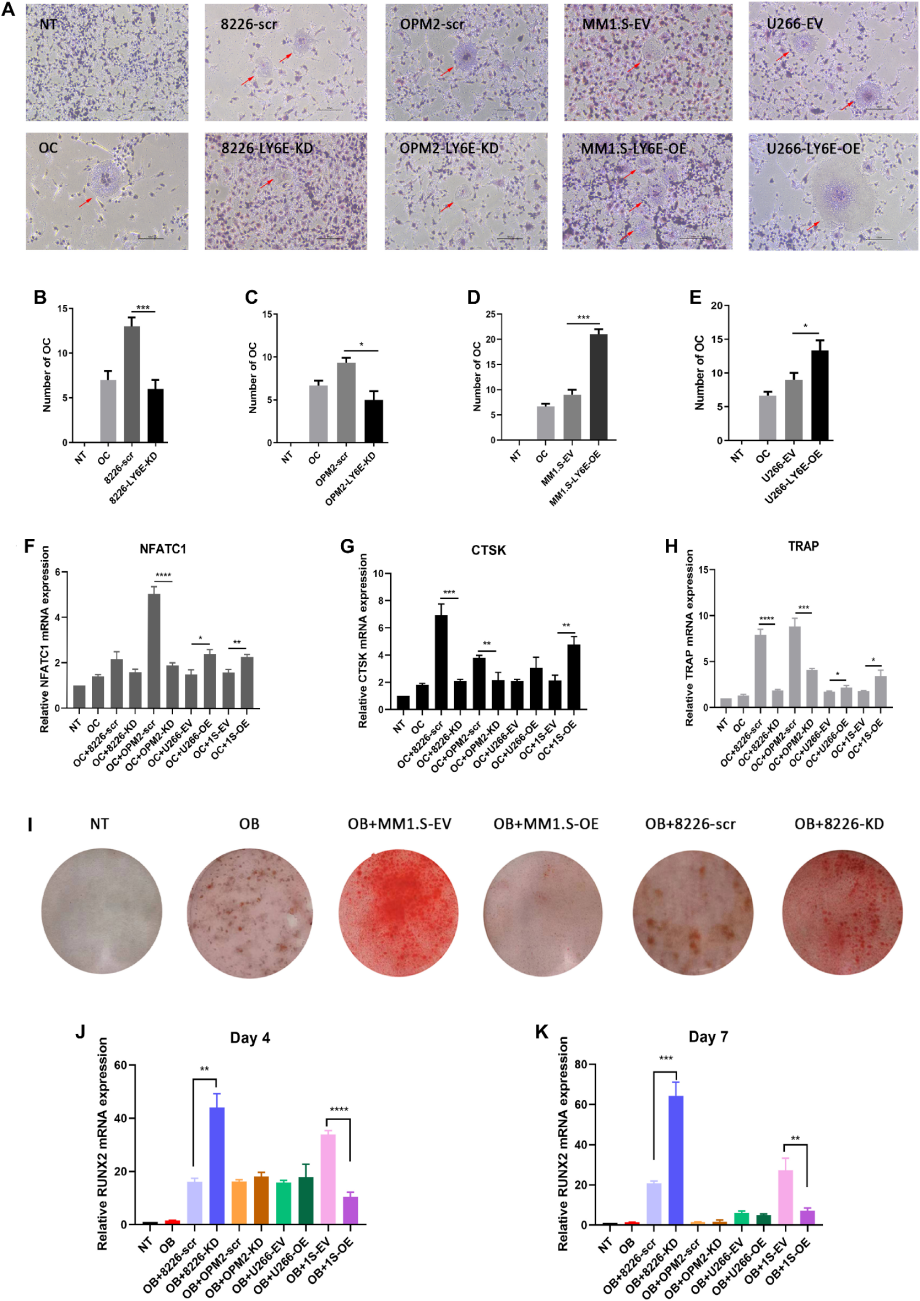
The diverse role of LY6E on osteoclasts and osteoblasts differentiation. (**A**) TRAP staining revealed that LY6E accelerated osteoclast differentiation (red arrows) when RAW264.7 macrophages were cultured with DMEM and CM. (**B**-**E**) Quantitated multinucleated osteoclasts. (**F**-**H**) NFATC1, CTSK, and TRAP expression in RAW264.7 cells. (I) MC3T3-E1 cells cultured in osteogenic medium and CM. Alizarin red S staining reveals the osteoblast differentiation at day 21. (**J**-**K**) qRT-PCR detected the expression of RUNX2 at day 4 and day 7. OB: osteoblast. OC: osteoclast. * *P* < 0.05; ** *P* < 0.01; *** *P* < 0.001.

To ascertain whether the role of LY6E in the formation of bone lesions, the osteoclast medium was utilised as an incubator for RAW264.7 cells. Notably, osteoclast formation was detected by TRAP staining on d5 (Fig. 6A). TRAP staining and relative counting analysis of multinucleated osteoclasts demonstrated that LY6E overexpression enhanced osteoclast differentiation when treated with RANKL (15ng/mL). Nevertheless, LY6E knockdown was observed to inhibit osteoclast differentiation (Fig. 6B-E). Furthermore, the expression of TRAP, CTSK, and NFATC1 mRNA was detected in RAW264.7 cells on d4 (Fig. 6F-H). The data obtained from qRT-PCR revealed that LY6E overexpressed cells memorably stimulate three genes mRNA expression in RAW264.7 cells. LY6E inhibited osteogenic activity in RPMI-8226 and MM1.S cells but this phenomenon was not replicated in OMP2 and U266 cells (data not shown), whether Alizarin Red S staining (Fig. 6I) or Runx2 mRNA expression (Fig. 6J-K) was considered. This may be attributed to the diverse osteoblastic phenotypes exhibited by different MM cells.

## Discussion

MM prognostic risk models incorporating sociodemographic, immunophenotypic, and clinical characteristics have been extensively studied. While the pathology of MBD has been well documented, the specific mechanism of bone injury remains a topic for further investigation. Prior studies have highlighted the significance of MBD, particularly in terms of its impact on patient outcomes and quality of life^28^. Consequently, the objective of this study was to ascertain the value of target gene related to bone disease and prognosis in MM.

LY6E, a member of Ly6/uPAR family^29,30^, is located on human chromosome 8q24.3 and mouse chromosome 15^31,32^. The LY6/uPAR superfamily comprises a variety of LY6 proteins, the glycoprotein CD59, and the lipoprotein-binding protein GPIHBP1. *LY6E* is composed of 8 to 10 cysteines forming a highly conserved three-finger folded motif via disulfide bonds^33^. The function of *LY6E* in the glycosylphosphatidylinositol (GPI) anchor of cell membrane proteins is of great importance in cell signal transduction, immune regulation, virus infection, tumor metastasis and cellular adhesion^34,35^.

LY6E has been demonstrated to exhibit transcriptional activity in a number of organs and tissues such as the lung, liver, uterus, myeloid cells, and thymocytes. Additionally, it is highly expressed in various tumors such as breast, stomach, esophagus, and colorectal^36^. Tang et al.^37^ demonstrated that LY6E may serve as a potential marker for assessing lupus activity. Meanwhile, the recent case also reported that LY6E was overexpressed in human pancreatic cancer stem cells and the high-level expression of LY6E may potentially contribute to tumorigenic^38^. In addition, Wang et al.^39^ demonstrated that the increased level of LY6A/E expression in enterococcus faecalis-infected murine colon epithelial cells resulted in oncogenic transformation. Yeom and other studies have identified LY6E as one of HIF-1 agonists, with abnormal overexpression increasing HIF-1α expression at the transcriptional level^40^. Ria et al. implied that HIF-1a may induce angiogenesis and promote bortezomib and lenalidomide resistance in multiple myeloma endothelial cells.

Currently, few studies have been published concerning an association between LY6E and MM bone destruction. Here, we finally obtained the multiple myeloma-related bone disease gene *LY6E* through a series of cohorts based on the DEGs of MM datebases. To verify the prognostic value of LY6E in MM, the patients were classified into LY6E-high and LY6E-low groups. In GSE24080, GSE2658, and GSE9782 databases, the LY6E-low group was significantly associated with improved survival outcomes according to the result of the K-M survival curve. This finding was corroborated by the bone marrow IHC and prognostic results patients at our institution.

The occurrence of osteolysis in MM is linked to a reduction in osteoblast production and an increase in osteoclast production^41^. In our study, the biological markers in osteoclasts were consistent with the high expression of LY6E, suggesting that it was related to osteoclast formation. Subsequently, we investigated the association between LY6E expression and clinicopathological indicators, and demonstrated the predictive performance of LY6E expression as an independent variable. Noteworthy,

A comparison of the GO enrichment and KEGG pathway results revealed an intriguing phenomenon: an apparent correlation between interferon-gamma (IFN-γ) and high bone patients in MM. In light of the PPI network results, it seems plausible to suggest that LY6E may be situated within the network of IFN-γ related family gene regulation. A considerable body of research has indicated that IFN-γ plays a dual role in osteoclast differentiation^42,43^. Specifically, low levels of IFN-γ have been shown to promote osteoclast differentiation, whereas high levels of IFN-γ have the potential to inhibit osteoclast formation. Furthermore, the outcome of upregulated DEGs in KEGG pathway enrichment was correlated with the Wnt signaling pathway, which played a fundamental role in the growth and differentiation of osteoblasts^18^. Nevertheless, the precise mechanism remains elusive and requires further investigation through additional experimental trials. In summary, LY6E may serve as a potential biomarker for the diagnosis and prognosis of MM patients with bone disease.

## Conclusion

Consequently, this study preliminarily analyzed the role of LY6E in MM with bone lesions and prognosis. Our findings identified that LY6E participated in the biological process of MM and MBD formation. Increased LY6E expression promotes the progression of MM disease and deteriorated survival. Further research is required to gain a deeper understanding of the signal network in physiology and disease, analyze LY6E gene expression regulation, identify upstream and downstream targets, and gain a more comprehensive understanding of LY6E biology. This will provide the basis for the development of tumor-targeted therapy.

## Electronic supplementary material

Below is the link to the electronic supplementary material.

Supplementary Material 1

## Data Availability

No datasets were generated or analysed during the current study.
