# Supplementary material for: LY6E as a new prognostic biomarker of multiple myeloma-related bone disease
Source: Sci Rep. 2025 Apr 3;15:11431. doi: 10.1038/s41598-025-91413-1 (PMC11968821; doi:10.1038/s41598-025-91413-1)
Supplement: Supplementary file 1 — Supplementary Material 1 [file 41598_2025_91413_MOESM1_ESM.pdf]

Supplemental information

A

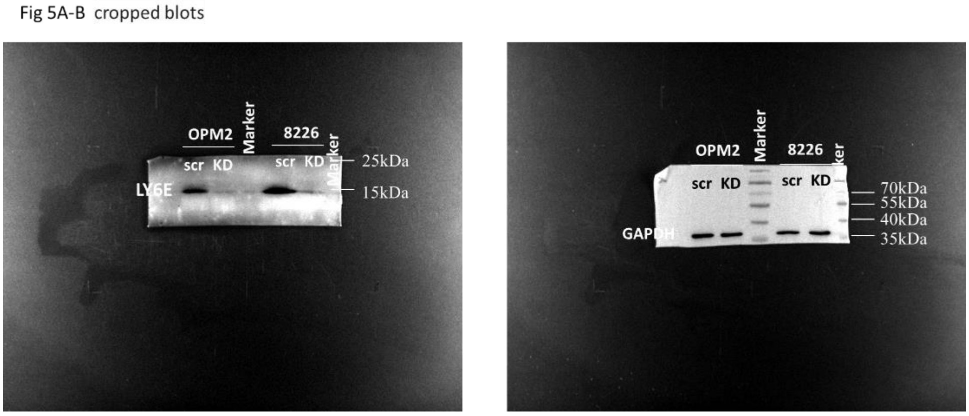

B

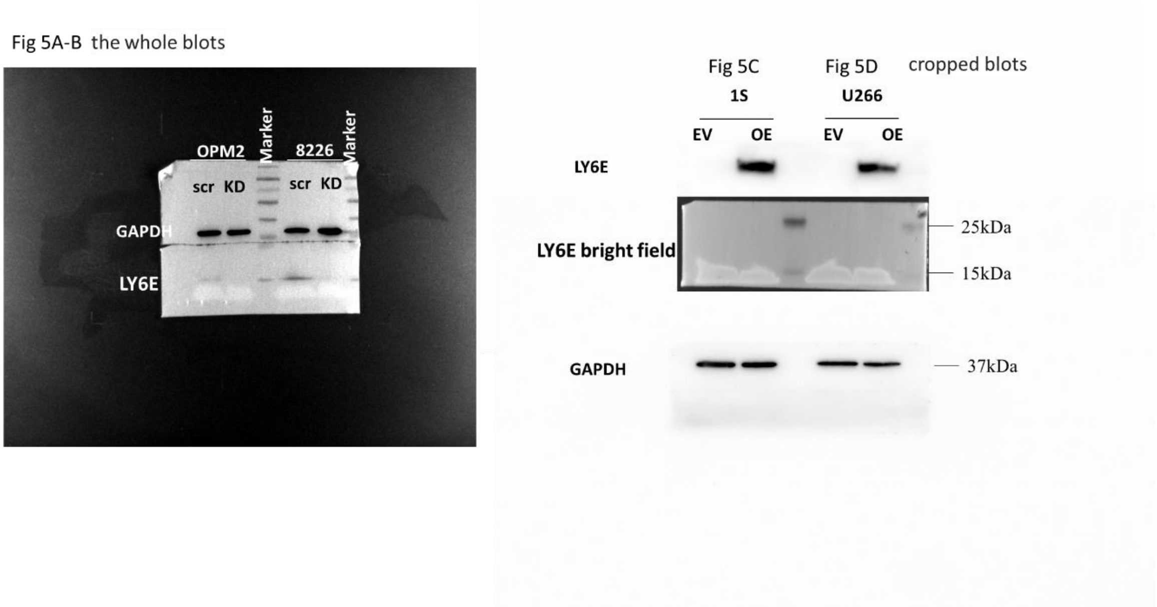

Figure S1. Original blots validating the expression of LY6E in Figure 5. (A) figure 5 A-B cropped blots. (B) figure 5 A-B the whole blots and figure 5 C-D cropped blots.

Table S1. Primers for the qRT-PCR.

Name	Sequence
GAPDH_F	AAGCCCATCACCATCTTCCA
GAPDH_R	CCTGCTTCACCACCTTCTTG
TRAP_F	ACACAGTGATGCTGTGTGGCAACTC
TRAP_R	CCAGAGGCTTCCACATATATGATGG
CTSK_F	AGCAGGCTGGAGGACTAAGGT
CTSK_R	GATTTGTGCATCTCAGTGGAAGAC
NFATC1_F	CCTTATGTGGCTCAGGTCTTACTTC
NFATC1_R	TGGTCCCCGAGACCACAAT

Table S2. The Univariate Cox of 5 bone associated genes in GSE24080.

Variables	OS			EFS		
	HR	95%*CI*	*P*	*HR*	95%*CI*	*P*
LY6E	1.12	1.04-1.20	<0.01	1.08	1.02-1.15	<0.01
BMPR1A	0.99	0.89-1.10	0.84	0.97	0.89-1.07	0.56
CSF2RB	1.00	0.91-1.11	0.93	0.93	0.86-1.01	0.07
CST6	0.97	0.91-1.03	0.31	0.97	0.92-1.01	0.17
ADTRP	0.95	0.89-1.02	0.16	0.93	0.88-0.98	<0.01

Table S3. Univariate and multivariate Cox regression analyses for EFS in GSE24080

Variables	Univariate model			Multivariate model		
	HR	*95%CI*	*P*	*HR*	*95%CI*	*P*
Sex	1.01	0.78-1.30	0.94			
Age ≥ 65y	1.42	1.06-1.89	0.02	1.24	0.92-1.67	0.16
Race	0.68	0.44-1.05	0.08			
B2M ≥ 3.5mg/L	1.90	1.48-2.44	<0.01	1.42	1.03-1.95	0.03
CRP ≥ 4mg/L	1.29	1.00-1.66	0.05	1.10	0.85-1.43	0.48
CREAT ≥1.2mg/dL	1.47	1.14-1.9	<0.01	1.06	0.795-1.42	0.68
LDH ≥ 170U/dL	1.86	1.45-2.39	<0.01	1.66	1.28-2.61	<0.01
ALB ≥ 3.5g/dL	0.67	0.48-0.93	0.02	0.75	0.53-1.07	0.11
HGB > 100g/L	0.64	0.49-0.82	<0.01	0.82	0.61-1.10	0.19
LY6E high expression	1.73	1.33-2.25	<0.01	1.71	1.31-2.24	<0.01

Abbreviations: B2M: β2-Microglobulin; CRP: C-reactive protein; CREAT: Serum creatinine; LDH: Lactate Dehydrogenase; ALB: Serum Albumin; HGB: Haemoglobin.

Table S4. Univariate and multivariate Cox regression analyses for OS in GSE24080

Variables	Univariate model			Multivariate model		
	HR	*95%CI*	*P*	*HR*	*95%CI*	*P*
Sex	1.03	0.76-1.40	0.84			
Age ≥ 65y	1.37	0.97-1.94	0.07			
Race	0.94	0.59-1.52	0.81			
B2M ≥ 3.5mg/L	2.15	1.59-2.92	<0.01	1.57	1.08-2.28	0.02
CRP ≥ 4mg/L	1.54	1.13-2.09	0.01	1.25	0.90-1.72	0.18
CREAT ≥1.2mg/dL	1.73	1.28-2.34	<0.01	1.21	0.87-1.70	0.26
LDH ≥ 170U/dL	2.19	1.62-2.96	<0.01	1.91	1.39-2.62	<0.01
ALB ≥ 3.5g/dL	0.52	0.36-0.76	<0.01	0.60	0.41-0.87	0.01
HGB > 100g/L	0.67	0.49-0.91	0.01	0.95	1.41-2.64	0.79
LY6E high expression	1.91	1.4-2.6	<0.01	1.93	1.41-2.64	<0.01

Abbreviations: B2M: β2-Microglobulin; CRP: C-reactive protein; CREAT: Serum creatinine; LDH: Lactate Dehydrogenase; ALB: Serum Albumin; HGB: Haemoglobin.

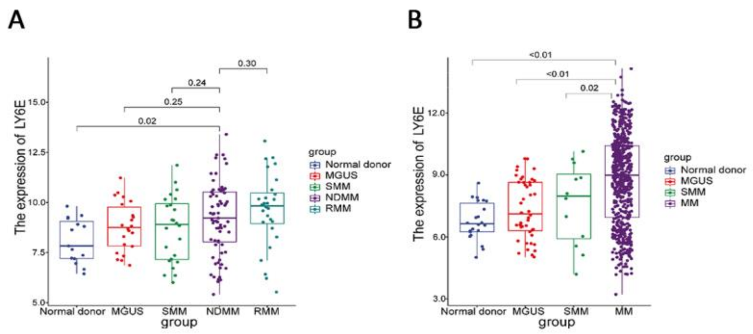

Figure S2. LY6E expression level in different populations. (A) A box plot shows LY6E expression of 5 groups (normal donor, MGUS, SMM, NDMM and RMM) in GSE5900 and GSE2658. (B) LY6E expression of 4 groups (normal donor, MGUS, SMM, and MM) in GSE6477. MGUS: monoclonal gammopathy of undetermined significance; SMM: smoldering multiple myeloma; NDMM: new diagnosed multiple myeloma; RMM: relapse MM.
